# Strongly sexually dimorphic forelegs are not more condition-dependent than less dimorphic traits in *Drosophila prolongata*

**DOI:** 10.1007/s10682-022-10226-0

**Published:** 2023-01-09

**Authors:** Jhoniel Perdigón Ferreira, Patrick T. Rohner, Stefan Lüpold

**Affiliations:** 1 Department of Evolutionary Biology and Environmental Studies, University of Zürich, Winterthurerstrasse 190, 8057 Zürich, Switzerland; 2 Department of Biology, Indiana University, 915 East Third Street, 102 Myers Hall, Bloomington, IN 47405 USA

**Keywords:** Secondary sexual traits, Trait exaggeration, Mating success, Allometry, Sexual selection

## Abstract

Directional sexual selection drives the evolution of traits that are most closely linked to reproductive success, giving rise to trait exaggeration and sexual dimorphism. Exaggerated structures are often costly and, therefore, thought to be expressed in a condition-dependent manner. Sexual selection theory thus predicts a direct link between directional sexual selection, sexual dimorphism, and sex-specific condition dependence. However, only a handful of studies investigate the relationship between sexual dimorphism and condition dependence. Using 21 genetic lines of *Drosophila prolongata*, we here compared the degree of sexual dimorphism and sex-specific condition dependence, measured as allometric slopes, in sexually selected and non-sexual traits. Our data revealed male-biased sexual dimorphism in all traits examined, most prominently in the sexually selected forelegs. However, there was no relationship between the degree of sex-specific condition dependence and sexual dimorphism across traits and genetic lines. Our results contradict theoretical predictions and highlight the importance of understanding the role of exaggerated traits in the context of both sexual and natural selection.

**Supplementary Information:**

The online version contains supplementary material available at 10.1007/s10682-022-10226-0.

## Introduction

Sexual selection is predicted to favor the exaggeration of pre- and post-mating traits whenever the extent of their elaboration increases reproductive success through male-male competition or female choice (Darwin 1871; West-Eberhard 1983; Andersson 1994; Emlen 2008; Lüpold et al. 2016). Since sexual selection is stronger on males than on females in most species (Bateman 1948; Trivers 1972; Clutton-Brock and Parker 1992), traits acting as ornaments or armaments often evolve absolutely or relatively greater expression in males relative to females (i.e. male-biased sexual dimorphism). However, trait elaboration is typically costly in terms of energy and fitness (e.g., Somjee et al. 2018; Rometsch et al. 2021), and a male’s resource allocation to sexual traits —and thereby the net fitness benefits gained— depends on his somatic, genetic, or epigenetic condition. Condition dependence is a form of developmental plasticity where an individual’s available metabolic resources determine the extent of trait expression by optimizing the relative resource allocation between somatic maintenance and reproduction (Nur and Hasson 1984; Andersson 1986; Rowe and Houle 1996; Hill 2011). Although any trait has the potential to vary with condition, sexually selected traits are predicted to be particularly sensitive to condition due to their diversion of resources from somatic maintenance and survival (Rowe and Houle 1996; Cotton et al. 2004).

With stronger sexual selection on males than on females, resulting in the evolution of male-biased sexual dimorphism in condition-dependent traits, it follows that condition dependence itself should be sexually dimorphic. In other words, variation among males in the expression of sexually selected traits should be more tightly linked to their bearers’ underlying condition than that among females. Consequently, traits that differ more between the sexes should also show greater divergence in sex-specific condition dependence than less dimorphic ones, mediated by sex-specific resource diversion from somatic maintenance and differential viability costs (Rowe and Houle 1996). Even though theory predicts such links between sexual selection, sexual dimorphism and condition-dependent trait expression, however, only few empirical studies have integrated some of these predictions (Bonduriansky 2007a, b; Oudin et al. 2015; Miller et al. 2016; Rohner and Blanckenhorn 2018).

In addition to the paucity of studies exploring condition dependence of traits in the context of sexual dimorphism, other limitations in interpreting the link between sexual selection and condition dependence are also prevalent. Specifically, many studies provide correlational rather than experimental evidence of condition dependence, and they are often limited to a focal trait without appropriate control traits or accounting for variation in body size (reviewed in Cotton et al. 2004; but see, Rohner and Blanckenhorn 2018; Fox et al. 2019; Cattelan et al. 2020, for some recent examples addressing these issues). Stronger and less biased evidence can come from comparisons across multiple traits that inform about their relative condition dependence within the same set of individuals, thereby placing the trait of interest in the context of general trait variation (Arnqvist and Thornhill 1998; Bonduriansky and Rowe 2005; Fairbairn 2005; Rohner and Blanckenhorn 2018). Additionally, since individuals can vary in their sensitivity to developmental stress, in their efficiency in turning acquired resources into growth and in their resource allocation strategy, studying condition dependence in a genetic context seems particularly important for sexually selected traits, in which trait elaboration is often assumed to signal genetic quality (Iwasa et al. 1991; Rowe and Houle 1996). Yet, the genetic contribution to condition dependence is rarely studied (but see, David et al. 2000; Kemp and Rutowski 2007; Hubbard et al. 2015).

In insects, as in many other taxa, the relative size of sexual traits is strongly influenced by the resource availability during juvenile development (David et al. 2000). In cyclorrhaphan Diptera, adult structures grow mostly during late larval and pupal development. Because late third-instar larvae and pupae cannot acquire more energy by feeding, the developmental precursors underlying different adult tissues develop in an energetically closed system wherein they directly compete for resources (Nijhout and Emlen 1998; Heming 2018). In adults with juvenile development under different nutritional conditions, shifts in relative resource allocation to trait growth directly relate to the dependency of trait expression on resource availability, i.e. condition (Rohner and Blanckenhorn 2018; Shingleton and Frankino 2018). To the extent that condition covaries with body size due to the greater metabolic resource pool available to larger pupae (Blanckenhorn 2000), larger individuals may be able to allocate relatively more resources to a fitness-enhancing trait (e.g., sexual ornament) before experiencing a viability cost compared to smaller individuals (Bonduriansky and Day 2003). If so, that condition-dependent fitness trait would scale disproportionately (i.e., positively allometrically) with body size across individuals (Bonduriansky and Day 2003). Studying nutrition-dependent trait expression using static allometries (i.e., the degree to which trait size changes with overall body size) thus permits testing whether trait exaggeration and sexual dimorphism relate to (sex-specific) condition dependence, and how the expression of one trait depends on the investment in others.

**Fig. 1:**
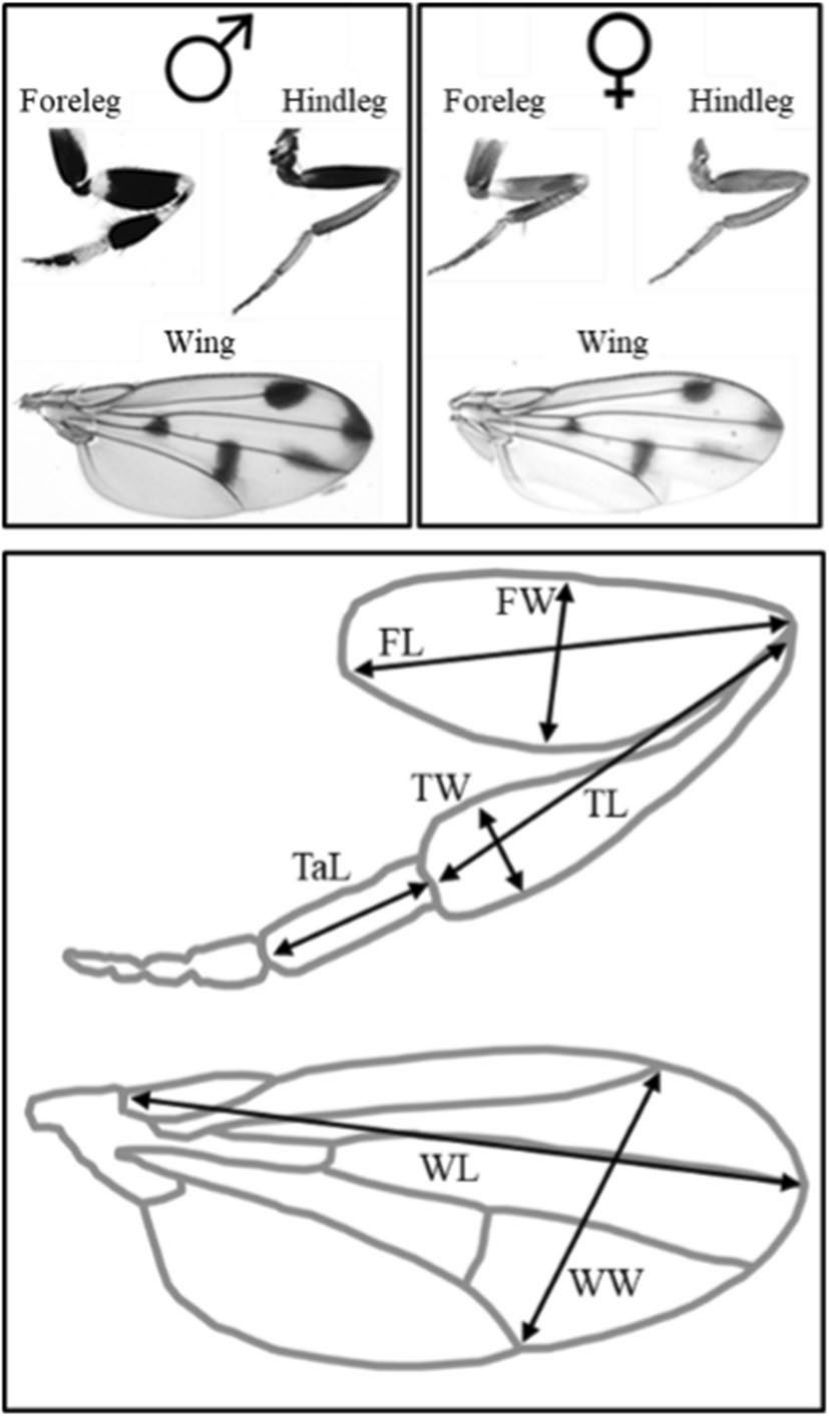
Morphology of the foreleg, hindleg, and wing of male and female *Drosophila prolongata*. The lower panel illustrates the linear measurements, using the male foreleg and wing diagram as examples. FL: femur length; FW: femur width; TL: tibia length; TW: tibia width; TaL: tarsus1 length; WL: wing length; WW: wing width. Throughout this study, these abbreviations for leg components are preceded by the leg (fore- or hindleg; e.g., FFL for forefemur length, HTW for hindtibia width)

An ideal system to study how sexual selection drives sex-specific condition dependence is the fruit fly *Drosophila prolongata*. As an exception within the Drosophilidae, *D. prolongata* males are much larger than females (Kudo et al. 2015; Rohner et al. 2018a, b) and develop dramatically enlarged forelegs with conspicuous black and white stripes (Setoguchi et al. 2014; Fig. 1). Males use these forelegs to strike their opponents in dyadic fights (Kudo et al. 2015), and to wave at, or occasionally stimulate the abdomen of, the female during courtship (Setoguchi et al. 2014, 2015; Perdigón Ferreira and Lüpold 2022). These functions, combined with the male-biased sexual dimorphism in foreleg expression and overall body size, suggest intense premating sexual selection on males, with forelegs as a primary target. For example, Perdigón Ferreira and Lüpold (2022) showed that males stimulating the female’s abdomen during courtship through “leg vibration” (Setoguchi et al. 2014, 2015) had significantly higher mating success compared to males that did not show this behavior.

Here, using *D. prolongata* isofemale lines, we investigated the link between sexual dimorphism and sex-specific condition dependence (i.e., static allometries) in a genetic context. Overall, we tested the hypothesis that traits under sexual selection in males (e.g., exaggerated forelegs) show higher levels of male-biased sexual dimorphism and condition dependence than other traits (e.g., hindlegs not involved in mating behavior). Rather than focusing solely on total leg size, we also tested for differential patterns of condition dependence in different parts of the leg to infer a possible role of any such part in the unique male behaviors.

## Materials and methods

### Study organism

*Drosophila prolongata* is a member of the rhopaloa subgroup within the melanogaster species group, with a geographic distribution that includes southwestern China, northeastern India, Myanmar and Vietnam (Singh and Gupta 1977; Toda 1991; Setoguchi et al. 2014). For this study, we used flies from 42 isofemale lines that were originally collected in their natural habitat near Sa Pa (22°20’N, 103°52’E), Vietnam, in 2004 and 2015 by H. Takamori (Kudo et al. 2015), and in 2018 by J.P.F. (Perdigón-Ferreira and Lüpold 2022). In order to avoid variation across our genotypes that might result from differential inbreeding depression (e.g., due to deleterious recessive alleles; Wright et al. 2008), we crossed males of one isofemale line with virgin females of another in 21 independent pairwise combinations (i.e., using each isofemale line only once). Throughout our experiments, we used these heterozygous F_1_ genotypes (henceforth referred to as ‘lines’). We maintained all larvae and adult flies in a climate chamber on a 14:10 light:dark cycle at 18 °C and 60% humidity.

### Manipulation of condition through changes in larval diet

To evaluate the condition dependence of trait expression, we manipulated the amount of food available to each developing larva. To this end, we first allowed adult flies of each pair of parental lines to feed and oviposit on standard fly food medium (replaced daily). Across 7 consecutive days, we then collected up to 600 first-instar larvae from each line and transferred them in groups of 50 to culturing vials with 3 different nutrient dilutions (each across 4 replicate vials per line and diet). The high-condition diet (“high condition” or “H”) contained 13 g of standard fly food (consisting of 55 g corn, 80 g agar, 100 g flour, 75 g glucose, 100 g fresh yeast, 10ml Nipagin antimicrobial agent per liter of food medium). We then diluted this standard food medium with water and agar to the same consistency but containing either half (“medium condition” or “M” diet) or one fifth (“low condition” or “L” diet) of the original nutrients in the same volume of medium. Towards the end of immature development, we checked all vials and tubes daily, and collected, counted, and froze all newly emerged individuals for later measurements.

### Morphometric measurements

For each male and female fly, we carefully removed with forceps the left foreleg, hindleg, and wing, and mounted them in Euparal between a glass microscope slide and a coverslip. We measured thorax length (distance between the tip of the scutellum and the base of the head, lateral view) of all individuals to the nearest 25 μm using a Leica MS5 stereomicroscope with an ocular reticle. This measurement was used as an estimate of body size, because it scales nearly isometrically with body weight and is a widely used proxy of body size in the *Drosophila* literature (e.g., Rohner et al. 2018a). We then captured photos of all appendages using a Leica M205 C stereoscope with an ORCA-Flash4.0 LT + Digital CMOS camera C11440 attached to it. Based on landmarks digitized using tpsDig2 version 2.32 (Rohlf 2016), we measured the lengths of the femur, tibia, and first tarsal segment of the fore- and hindleg, respectively (for measurements and abbreviations see Fig. 1). We further measured the maximal widths of the femur and tibia of each leg, as well as the length and width of the wing (Fig. 1).

### Statistical analyses

To test for the diet treatment effect (hereafter referred to as “treatment”) on the larval survival rate, we used a generalized linear mixed-effects model (GLMM) with a binomial error distribution, using vial nested within line as random effect (hereafter, “line/vial” random effect) to control for the non-independence of flies within lines and vials during development. We further used linear mixed-effects models (LMEs) to test for an effect of treatment and sex, as well as their interaction, on both egg-to-adult development time and adult body size, again using line/vial as a random effect. The analysis on body size was done using log-transformed thorax length as the response variable. We also tested for the effects of our treatments on the relative size of all focal body parts by performing separate LMEs including thorax length (our estimate of body size) as a covariate and the line/vial random effect. To estimate the contribution of the random effect to the total variance, we compared models with and without the line/vial random effect in likelihood ratio tests (LRTs). Throughout, we focused only on random intercepts due to much poorer performance of random-slope analyses in model comparisons.

To estimate sex-specific condition dependence, we followed the approach described in Rohner and Blanckenhorn (2018). Instead of analyzing the effect of the three treatments as discrete categories, we considered their effect on body and trait size as continuous and estimated sex-specific static allometries. We restricted these analyses to the H and L individuals to capture the full range of body sizes. In addition, this allowed us to keep the M individuals for estimates of sexual dimorphism (see below) to avoid spurious correlations between condition dependence and sexual dimorphism by using partially overlapping individuals between sexes and treatments (Oudin et al. 2015). In brief, we log-transformed all trait values and then calculated sex-specific allometric slopes of all traits against body size. This means that we obtained 42 slopes for each trait (i.e., one slope for each sex and line). To test for deviations from isometry (*β* = 1) in the sex-specific relationship between trait size and body size, we performed ordinary least squares (OLS) regressions using the *sma()* function of the R package *smatr* version 3.4.8 (Warton et al. 2012), which includes the *robust* option that controls for the possible effect of outliers on the slope estimation inference. Even though allometric relationships are often calculated by standardized (or reduced) major-axis regressions, OLS regressions have recently been shown not to underestimate slopes as previously thought, and to be less sensitive to extreme values (Al-Wathiqui and Rodríguez 2011; Kilmer and Rodríguez 2017). We then tested whether the male and female trait-specific allometries were correlated across lines by performing an LME with trait and line as random effects. Noteworthy, the *sma()* function does not allow the inclusion of random effect impeding us from controlling for possible vial effects. We again tested for a possible contribution of line to the total variance using likelihood ratio tests. Further, we used the logarithm of the ratios of male over female allometric slopes [i.e., log(male slope) – log(female slope)] to estimate the trait-specific sex differences in condition dependence, resulting in 21 indices of condition dependence for each trait.

To calculate sexual dimorphism, we used the flies originating from treatment “M”. We first removed any trait variation due to overall body size by calculating the residual trait sizes derived from LMEs of the log-transformed focal trait size against thorax length across both sexes combined, with line/vial as a random effect. We then *z*-transformed these residuals across sexes before averaging them within each sex. The trait-specific mean difference between sexes ($\stackrel{-}{x}$_males_ − $\stackrel{-}{x}$_females_) represents the index of relative trait size dimorphism. Here too, we obtained one index of sexual dimorphism per line (i.e., 21 values per trait). We then tested if these values were associated with the trait-specific sex differences in condition dependence (above) in an LME with trait as random effect. Again, we tested for the significance of the line random effect using an LRT. We conducted all statistical analyses in R v.4.0.4 (R Core Team 2021).

In summary, we tested for the degree of sexual dimorphism, condition dependence, and their relationship across 12 morphological traits. Based on observations of male courtship and fighting behavior (Perdigón Ferreira and Lüpold 2022), we predicted male forelegs to be the most sexually dimorphic trait, with wings and hindlegs showing intermediate and low levels of dimorphism, respectively. In addition, we expected male forelegs, a trait putatively under directional sexual selection (Setoguchi et al. 2014, 2015), to show the steepest allometric slopes when compared to all other traits (see Bonduriansky and Day 2003). Finally, we predicted a positive correlation between the degrees of condition dependence and sexual dimorphism across traits (Bonduriansky 2007a).

## Results

### Effect of larval food limitation on survival, development, and body size

We obtained 2061 adult males (1000 H, 741 M, and 320 L) and 1,685 adult females (797 H, 548 M, and 340 L). In a binomial GLMM, larvae reared under the H treatment survived better than those subjected to food limitation (M and L treatments) (H: mean (95% CI) = 46.5% (45.0–47.9); M: 34.3% (32.6–36.1); L: 16.9% (15.6–18.2); treatment effect; Wald *χ*^*2*^_*2*_ = 202.66, *P* < 0.001). The line/vial random effect was highly significant (LRT: *χ*^2^_2_ = 480.89, *P* < 0.001).

**Fig. 2:**
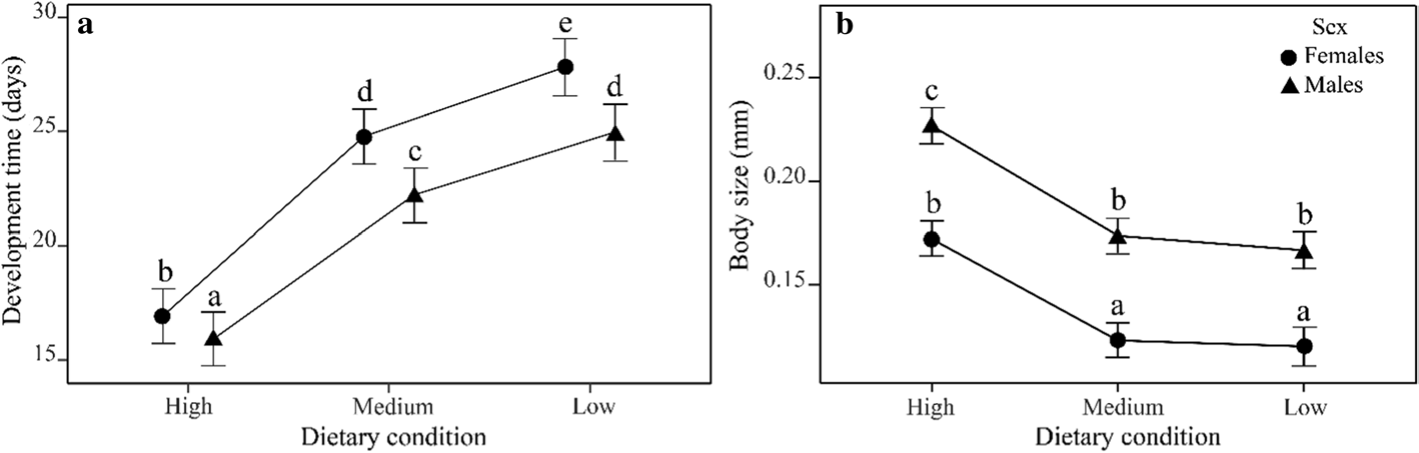
**A** Development time (from day of egg hatching to day of adult emergence) for males and females between high, medium and low nutrient concentration in the larval diet. **B** Body size (thorax length) for males and females across the same three treatments. Circles and triangles indicate the least-squares means, error bars their 95% confidence intervals. Means sharing a letter are not significantly different (*P* > 0.05; Sidak–adjusted comparisons)

An LME further revealed that the mean development time of the H treatment was 29% and 36% shorter compared to M and L ones, respectively (*F*_2,199.96_ = 226.78, *P* < 0.001; Fig. 2a). Females also developed faster than males in all treatments (*F*_1,204.15_ = 634.85, *P <* 0.001; females, H: mean = 16.7 (15.8–17.6); M: 22.1 (21.3–23.0); L: 24.5 (23.6–25.4) days; males, H: mean = 17.7 (16.8–18.6); M: 24.6 (23.8–25.5); L: 27.6 (26.7–28.5) days), and the response to the treatments was stronger in males than in females (treatment × sex interaction: *F*_*2,204.02*_ = 45.66, *P* < 0.001). Again, we found a significant line/vial random effect (LRT: *χ*^2^_2_ = 379.03, *P <* 0.001).

Finally, treatment had a significant effect on body size (Fig. 2b), with flies of the H treatment being larger than those of the M and L treatments (*F*_2,191.2_ = 176.47, *P* < 0.001). In addition, males were larger than females (*F*_1,1931.9_ = 1604.30, *P* < 0.001), and the treatment effect was stronger on males than on females (treatment × sex interaction: *F*_2,1924_ = 3.38, *P* = 0.034). Line/vial identity contributed significantly to the total variance in body size (LRT: *χ*^2^_2_ = 459.36, *P <* 0.001). We found similar treatment × sex interactions for the relative size of most leg and wing traits (Table 1).

**Table 1:** Least-squares means of the 12 traits for males and females reared in the three treatments, along with their *F*-values and significance levels

Trait	H		M		L		*F*-values		
	Males	Females	Males	Females	Males	Females	Sex	Treatment	Sex × Treatment
FFL	0.115	− 0.095	0.121	− 0.098	0.124	− 0.101	65,613.073***	0.777	47.197***
FFW	− 0.292	− 0.670	− 0.305	− 0.676	− 0.307	− 0.675	35,217.649***	9.893***	3.733*
FTL	0.112	− 0.128	0.115	− 0.133	0.117	− 0.139	89,474.860***	3.238*	48.498***
FTW	− 0.518	− 0.913	− 0.535	− 0.914	− 0.530	− 0.919	35,147.812***	5.715**	10.977***
FTaL	− 0.302	− 0.480	− 0.308	− 0.494	− 0.304	− 0.497	16,473.319***	18.996***	15.409***
HFL	0.005	− 0.049	− 0.008	− 0.049	− 0.006	− 0.053	1159.538***	10.084***	15.431***
HFW	− 0.582	− 0.646	− 0.608	− 0.652	− 0.610	− 0.654	324.639***	12.924***	10.893***
HTL	0.040	− 0.017	0.032	− 0.013	0.033	− 0.018	1324.016***	3.044*	12.028***
HTW	− 0.886	− 0.913	− 0.895	− 0.907	− 0.896	− 0.913	86.033***	1.335	5.355**
HTaL	− 0.275	− 0.361	− 0.293	− 0.362	− 0.291	− 0.368	1577.419***	13.429***	14.218***
WL	0.447	0.438	0.449	0.441	0.454	0.446	138.968***	24.207***	0.199
WW	0.102	0.089	0.103	0.092	0.107	0.094	183.644***	9.111***	2.410

### Condition dependence and sexual dimorphism

**Fig. 3:**
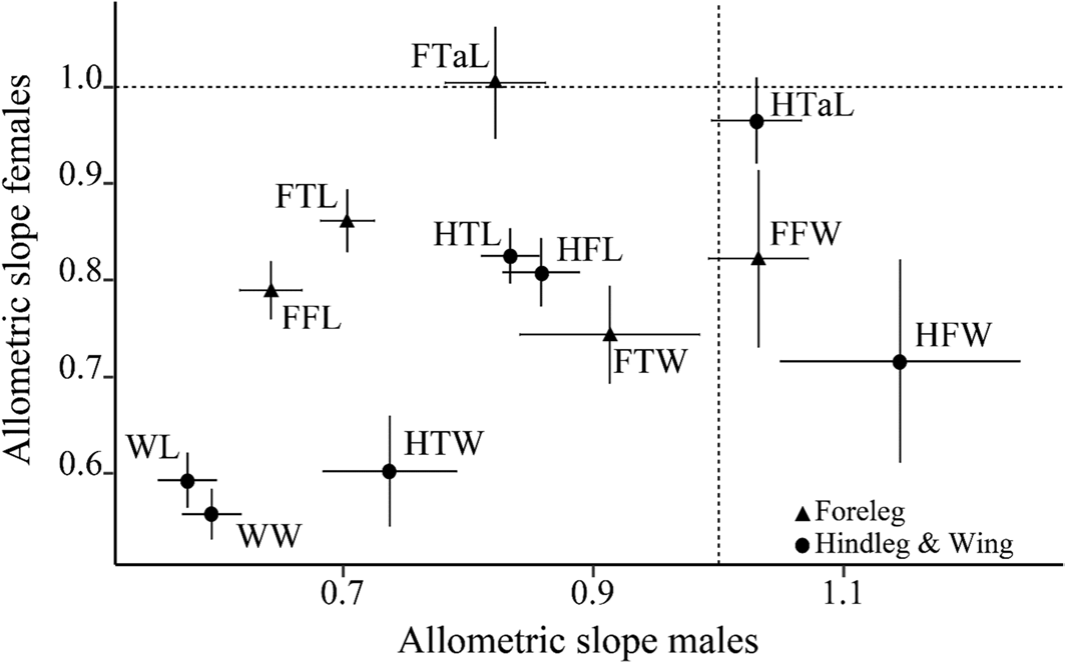
Relationship between static allometric slopes of males and females. Rectangles (foreleg traits) and dots (hindleg and wing traits) reflect means across the 21 lines with 95% confidence intervals. Horizontal and vertical dashed lines indicate isometry. Values above the horizontal and to the right of the vertical dashed lines indicate hyperallometry, and those below or to the left indicate hypoallometry. Traits labeled as in Fig. 1 except for being preceded by an F = foreleg or an H = hindleg

Independent of the sex, individuals of the 21 different genotypes showed similar allometric patterns within traits (Supplementary Table S1, Fig. S1). Out of 504 allometric slopes calculated, 252 were not significantly different from one (i.e., isometry), 194 shallower than one (i.e., hypoallometry), 23 were steeper than one (i.e., hyperallometry), and 35 were not significantly different from zero (Suppl. Table S1 and Fig. S1). Most traits that showed hyperallometric scaling in some lines (FFW, FTW, FTaL, HFW, and HTaL; for definitions see Fig. 1) did so in males but not in females (Suppl. Table S1). Overall, allometric slopes were not significantly correlated between the sexes (LME using trait and line identity as random effects: *F*_1,230.92_ = 2.87, *P* = 0.092), despite most lines showing a positive, albeit not always significant, relationship between male and female allometric slopes (Table 2). Among all traits, hindfemur width was the only one with a hyperallometric slope in all lines, at least for males, whilst all other traits were either hypo- or isometric in both sexes (Fig. 3). The line random effect explained a significant portion of the total variance (LRT: *χ*^2^_1_ = 71.15, *P <* 0.001).

**Table 2:** Across-trait Pearson’s product moment correlations between male and female OLS allometric slopes (sex-specific condition dependence, ssCD), and the sex difference in condition dependence and sexual dimorphism (SD) across the 12 morphological traits measured in each of the 21 lines

Line	ssCD			ssCD vs. SD		
	*t*	df	*r *[95% CI]	*t*	df	*r *[95% CI]
1	2.543	10	**0.627 [0.082, 0.883]**	− 0.432	10	− 0.135 [− 0.658, 0.476]
2	2.785	10	**0.661 [0.140, 0.895]**	− 0.468	10	− 0.146 [− 0.664, 0.467]
3	− 0.167	10	− 0.053 [− 0.608, 0.537]	− 1.050	10	− 0.315 [− 0.753, 0.316]
4	− 0.017	10	− 0.005 [− 0.578, 0.570]	− 0.997	8	− 0.332 [− 0.796, 0.376]
5	0.774	10	0.238 [− 0.389, 0.714]	− 1.306	10	− 0.381 [− 0.784, 0.2486]
6	0.505	10	0.158 [− 0.458, 0.671]	0.065	10	0.020 [− 0.560, 0.587]
7	0.956	10	0.290 [− 0.341, 0.740]	− 0.712	10	− 0.220 [− 0.705, 0.405]
8	2.451	10	**0.613 [0.060, 0.878]**	− 1.204	10	− 0.356 [− 0.772, 0.274]
9	− 2.215	10	− 0.574 [− 0.863, 0.000]	1.262	9	0.388 [− 0.276, 0.801]
10	2.709	10	**0.651 [0.122, 0.892]**	− 2.997	10	− 0.688 [− 0.904, 0.188]
11	1.529	10	0.435 [− 0.185, 0.808]	− 0.852	10	− 0.260 [− 0.726, 0.369]
12	1.433	10	0.413 [− 0.211, 0.798]	− 0.811	10	− 0.248 [− 0.720, 0.380]
13	0.993	10	0.300 [− 0.331, 0.745]	− 0.311	10	− 0.098 [− 0.636, 0.504]
14	5.470	10	**0.866 [0.580, 0.962]**	0.398	10	0.125 [− 0.484, 0.652]
15	4.596	10	**0.824 [0.474, 0.949]**	− 0.255	10	− 0.080 [− 0.625, 0.517]
16	− 0.445	10	− 0.139 [− 0.660, 0.472]	−0.789	10	− 0.242 [− 0.716, 0.385]
17	− 4.341	10	**− 0.808 [− 0.944, − 0.437]**	− 0.930	10	− 0.282 [− 0.737, 0.348]
18	2.637	10	**0.640 [0.105, 0.888]**	− 4.427	10	**− 0.814 [− 0.946, − 0.450]**
19	3.080	10	**0.698 [0.207, 0.908]**	0.128	10	0.041 [− 0.546, 0.601]
20	3.018	10	**0.690 [0.193, 0.906]**	− 0.816	10	-0.250 [− 0.720, 0.378]
21	2.403	10	**0.605 [0.048, 0.875]**	0.919	10	0.279 [− 0.351, 0.735]

Coefficients with 95% confidence intervals that do not overlap with zero are highlighted in bold

For lines 4 and 9, we were unable to calculate the condition dependence index (i.e. log(male slope – log(female slope)) for 2 and 1 trait, respectively, due to negative slopes

**Fig. 4:**
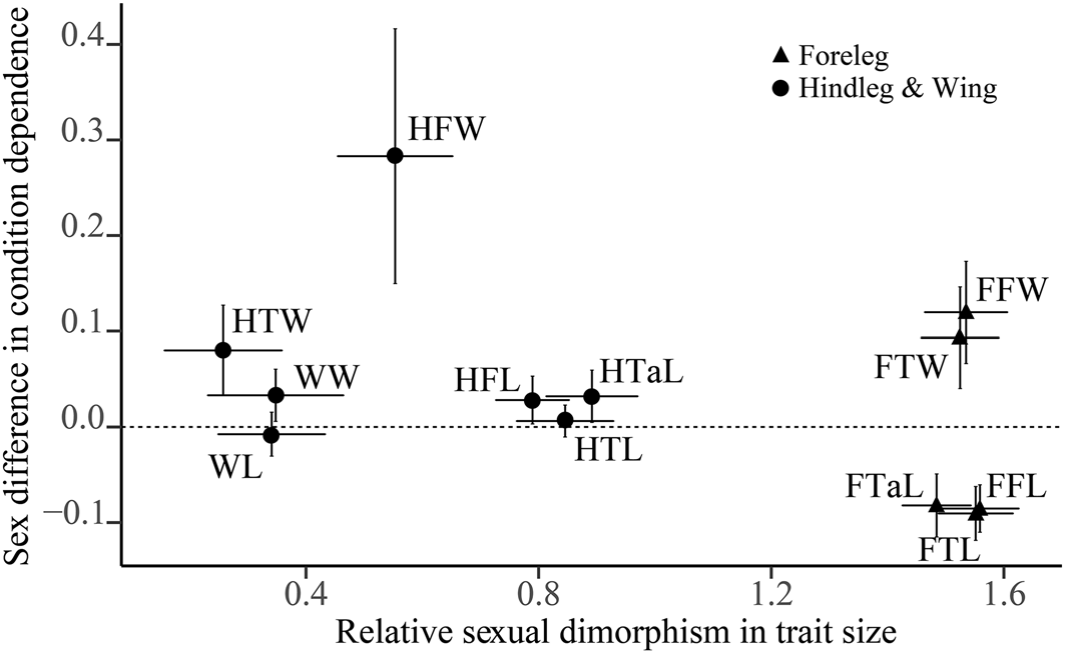
Relationship between sex-specific condition dependence (log(slope_males_) – log(slope_females_)) and relative sexual trait size dimorphism ($\stackrel{-}{x}$_males_ − $\stackrel{-}{x}$_females_). Rectangles (foreleg traits) and dots (hindleg and wing traits) are the trait-specific means across all 21 lines. Horizontal dashed lines indicate no difference in condition dependence between males and females and values above and below indicate male- and female-biased condition dependence, respectively. Note that all values on the *x-*axis are larger than zero, indicating male-biased sexual dimorphism. Error bars represent 95% confidence intervals. Traits labeled as in Fig. 1 except for being preceded by an F = foreleg or an H = hindleg

The relative sexual dimorphism of all traits considered was male-biased (Fig. 4). All foreleg parts measured showed the highest degree of sexual dimorphism whereas wing length and width, together with hindtibia width, were the least dimorphic. However, even though most traits were more condition-dependent in males, forefemur, foretibia and foretarsus lengths were significantly more condition-dependent in females compared to males (Fig. 4). In addition, the degree of relative sexual dimorphism was not correlated with the sex difference in condition dependence (linear mixed model using trait and line identity as random effects: *F*_175.46_ = 0.292, *P* = 0.591; Fig. 4). The significant line effect (LRT: *χ*^2^_1_ = 35.13, *P <* 0.001) suggests genetic variation in the strength of the relationship between the sex difference in condition dependence and sexual dimorphism. The lack of a relationship between the sex-specific trait expression and condition dependence was not the result of contrasting associations between lines that canceled one another but rather of no significant correlation in all but one line (Table 2).

## Discussion

Sexual selection theory predicts that traits under directional selection should be more sexually dimorphic and more condition-dependent than traits that are mostly under natural selection that acts similarly on both sexes (Rowe and Houle 1996; Cotton et al. 2004). Our results do not support the prediction that sex-specific trait exaggeration goes hand in hand with a sex-specific increase in condition dependence (Bonduriansky 2007b). Although the foreleg traits showed a high degree of sexual dimorphism in the predicted direction (i.e., male-biased sexual dimorphism), they were not more condition-dependent than other traits. That is, some foreleg parts that were more exaggerated in males were more condition-dependent in females, conflicting with theoretical predictions (Andersson 1986; Pomiankowski 1987; Rowe and Houle 1996). Below we discuss the evidence for directional sexual selection in male morphology, the relationship with sexual dimorphism and sex-specific condition dependence, and how it shapes trait covariation.

### Evidence of sexual selection acting on male foreleg and wing morphology

As in many drosophilids (e.g., Spieth 1974), wings are used in several courtship elements in *D. prolongata*, such as “unilateral wing vibration”, “bilateral wing vibration”, and “wing waving” (Setoguchi et al. 2014). Wing shape has been shown to affect mating success in male *D. melanogaster* (Menezes et al. 2013), and similar effects could also apply to *D. prolongata* (but see Perdigón Ferreira and Lüpold 2022). In addition to wings, male *D. prolongata* use their forelegs for repeated waving movements when courting females. This behavior is not limited to *D. prolongata*, but it is about 30 times more frequently observed than in closely related species (Setoguchi et al. 2014). In addition, male *D. prolongata* use their forelegs to stimulate the female abdomen with drumming movements after protracted courtship, possibly to increase the receptivity of reluctant females. Unlike all other drosophilids studied so far (e.g., Vedenina et al. 2013), however, male *D. prolongata* do not approach and court females from behind, but rather face the female and reach around her (Setoguchi et al. 2014; Perdigón Ferreira and Lüpold 2022). Consequently, this unusual way of stimulating the female abdomen could have played a pivotal role in the evolution of male foreleg exaggeration in this species (Setoguchi et al. 2014; Perdigón Ferreira and Lüpold 2022) either via directional (e.g. favoring males with larger legs) or stabilizing (e.g. favoring an average leg length) selection.

The effect of sexual selection on phenotypic traits can vary across ecological and social conditions (Miller and Svensson 2014; Evans and García-González 2016). Specifically, factors such as population density (Rittschof 2010; McCullough et al. 2018), sex ratio (Jann et al. 2000; Punzalan et al. 2010), or resource quality (Gillespie et al., 2014) can all change the strength and direction of sexual selection. Such effects might also play a role in *D. prolongata*. For example, in competitive mating trials using male duos, an outbred population, and fly food medium as the substrate, we found a mating advantage for the males that had developed under superior dietary conditions (and consequently were also larger in most cases; Perdigón Ferreira and Lüpold 2022). However, it is important to measure sexual selection in different contexts to better capture the naturally occurring fluctuations in sexual selection (Miller and Svensson 2014).

If fluctuations in the ecological and social context affect the strength and direction of sexual selection in *D*. *prolongata* males, this could explain the maintenance of phenotypic variation in body and trait size, despite the possible advantage of relatively larger males when fighting (Amino and Matsuo 2020) or courting (Perdigón Ferreira and Lüpold 2022). In addition, such fluctuations could have favored the evolution of interception (i.e., ‘stealing’ a female from a courting male) as an alternative reproductive tactic among relatively smaller males (Perdigón Ferreira and Lüpold 2022). In summary, assessing sexual selection under different and relevant ecological conditions can help to understand the circumstances that could have favored the evolution of the unique morphology and behavior of males and to better predict the type of allometric scaling (i.e., the level of condition dependence) shown by these sexually selected traits (Eberhard et al. 2018; McCullough and O’Brien 2022).

### Allometric scaling and sexual selection

Based on the striking sexual dimorphism in forelegs and on their role, together with the wings, in courtship, we predicted that these traits should show particularly marked differences in condition dependence between males and females. However, we found no clear evidence of a relationship between the trait-specific extent of sexual dimorphism and the sex difference in relative condition dependence (see Fig. 4).

Hyperallometric scaling, that is, a disproportionate increase in trait size relative to organismal body size that ultimately reflects condition-dependent trait expression, is often expected to be driven by strong directional selection (Bonduriansky and Day 2003). Here, most traits, even those predicted to be under directional sexual selection, were instead isometric or hypoallometric. Despite these apparent contradictions, our results are by no means exceptional, in that secondary sexual traits have previously been shown to scale hypoallometrically (Eberhard et al. 2018). In fact, it has been emphasized that the mode and strength of sexual selection, as well as different forms of natural selection, and possible genetic correlations between the sexes, must be considered when making predictions (e.g., Simmons and Tomkins 1996; Eberhard 2002; Fairbairn 2005; Eberhard et al. 2018; Kelly 2022; McCullough and O’Brien 2022; Palaoro et al. 2022). The forelegs of *D. prolongata*, for example, are also used for locomotion and might thus be more constrained or functionally integrated with variation in other traits (e.g., mid- and hindlegs) than traits in other species that are used in a sexual context only (e.g., Kelly 2014, 2022).

Like *D*. *prolongata*, males of the drosophilid *Chymomyza mycopelates* use their forelegs to rapidly flick and touch the female abdomen during courtship, and to either display at, or slam, their opponents in contests (Eberhard 2002). Despite clear indication that male forelegs function as both signals and weapons, the allometric slopes of all foreleg parts considered here were hypoallometric and comparable to those of the hindlegs that are not directly involved in courtship or fighting (see Katsuki et al. 2014, for a similar example in a beetle species). More recently, Kelly (2022) showed that the allometric relationships of leg and wing traits in the Japanese beetle *Popillia japonica* were shallower than unity, independently of whether sexual selection was directional or stabilizing. The same study also highlighted the importance of considering other types of selection (e.g., viability) when predicting allometric patterns. Thus, the different sexual and non-sexual functions of male forelegs complicate predictions about their allometric scaling (Bonduriansky 2007a), highlighting the importance of considering the context of trait use when predicting or comparing allometries, including different forms of use in a sexual context (functional weapon, coercion, intimidation, or courtship; Eberhard et al. 2018).

Finally, it is worth noting that our results differ from another study comparing the allometric slope of foreleg length between the sexes in the same species. In a reduced major-axis regression, Luecke and Kopp (2019) reported leg length to be isometric in both sexes, but males had higher mean trait values (i.e., shift in elevation). However, like Luecke and Kopp’s (2019) results, we found comparable allometric patterns between males and females, suggesting that the developmental constraints on the final foreleg size are not completely removed.

## Conclusion

Taken together, our results suggest that sexual dimorphism did not correlate with the degree of sex-specific condition dependence. Rather, our results illustrate that the relative importance of sexual selection in generating such patterns is likely to vary between species (e.g., Cotton et al. 2004) and to depend on the function of the trait (Eberhard et al. 2018). Moreover, even when theoretical models predict that these patterns should be common in secondary sexual traits, and empirical studies often find support thereof, there is nonetheless a notable proportion of traits that do not follow these patterns (e.g., Johnstone et al. 2009; Voje 2016). To better predict the relationship between exaggerated secondary sexual traits and other body parts among individuals, we need to better understand the role of such traits in the context of natural as much as sexual selection (e.g., Bro-Jørgensen et al. 2007). Only by investigating the costs and benefits of a multitude of fitness components can we better predict the outcome of selection acting on exaggerated secondary sexual traits.

## Electronic supplementary material

Below is the link to the electronic supplementary material.

Supplementary Material 1

## Data Availability

Analyses reported in this article can be reproduced using the data provided in Perdigón Ferreira et al. (2022).

## Code availability

The code use for the analysis of the data will be deposited on the Dryad data repository upon acceptance of the paper.
