# Supplementary material for: Strongly sexually dimorphic forelegs are not more condition-dependent than less dimorphic traits in Drosophila prolongata
Source: Evol Ecol. 2023 Jan 9;37(3):493–508. doi: 10.1007/s10682-022-10226-0 (PMC10156779; doi:10.1007/s10682-022-10226-0)

Supplementary material

Supplementary table S1. Sample size (*N*) and sex-specific static allometric slopes (β) and corresponding coefficients of determination (r^2). Allometries were calculated using ordinary least-squares regressions of log-transformed trait dimension against log-transformed thorax length. Results in bold are significantly different from 1 (isometry).

			Forefemur length				Forefemur width				Foretibia length				Foretibia width			
	N		r^2		β		r^2		β		r^2		β		r^2		β	
Line	♂	♀	♂	♀	♂	♀	♂	♀	♂	♀	♂	♀	♂	♀	♂	♀	♂	♀
1	30	31	0.885	0.948	**0.681**	**0.845**	0.917	0.525	1.121	0.777	0.863	0.908	**0.626**	**0.862**	0.775	0.742	0.967	0.837
2	23	11	0.801	0.917	**0.628**	**0.697**	0.856	0.430	0.857	1.083	0.800	0.947	**0.601**	**0.822**	0.579	0.542	1.074	0.727
3	30	43	0.824	0.829	**0.682**	0.855	0.928	0.363	1.080	1.117	0.923	0.820	**0.717**	0.930	0.704	0.363	0.981	0.529
4	37	40	0.702	0.266	0.671	**0.427**	0.800	0.000	0.942	**-0.148**	0.769	0.249	**0.636**	**0.424**	0.306	0.028	0.502	0.172
5	49	47	0.883	0.891	**0.785**	0.895	0.903	0.285	1.112	0.832	0.876	0.875	**0.744**	0.903	0.689	0.499	1.048	0.891
6	38	38	0.680	0.856	**0.514**	0.887	0.814	0.115	1.021	0.355	0.840	0.883	**0.706**	0.980	0.525	0.612	0.742	**0.708**
7	39	44	0.657	0.839	**0.714**	0.956	0.810	0.362	1.103	**0.628**	0.808	0.833	**0.718**	0.954	0.425	0.659	0.754	**0.749**
8	24	27	0.834	0.822	**0.632**	**0.723**	0.812	0.647	**1.191**	1.174	0.857	0.840	**0.732**	0.892	0.540	0.585	1.168	0.686
9	19	14	0.760	0.862	0.860	0.759	0.898	0.163	0.782	0.156	0.906	0.825	**0.719**	0.934	0.373	0.735	0.679	0.834
10	35	14	0.846	0.940	**0.588**	0.969	0.918	0.681	**1.202**	1.416	0.879	0.922	**0.638**	1.073	0.821	0.818	**0.821**	1.085
11	34	29	0.691	0.746	**0.674**	**0.724**	0.568	0.681	0.951	1.363	0.834	0.740	**0.693**	**0.793**	0.607	0.266	**1.624**	0.701
12	40	30	0.868	0.876	**0.679**	**0.800**	0.942	0.373	**1.322**	0.765	0.952	0.880	**0.860**	**0.712**	0.697	0.649	1.156	0.875
13	27	31	0.852	0.857	**0.615**	**0.743**	0.842	0.190	1.011	0.373	0.936	0.802	**0.722**	**0.765**	0.592	0.373	0.690	0.901
14	25	29	0.796	0.936	**0.473**	**0.731**	0.823	0.463	0.959	1.095	0.876	0.929	**0.729**	0.881	0.663	0.738	**0.531**	0.626
15	40	46	0.806	0.868	**0.695**	**0.890**	0.869	0.491	1.096	1.335	0.860	0.863	**0.757**	1.007	0.689	0.624	**1.287**	1.029
16	30	35	0.737	0.889	**0.714**	**0.825**	0.838	0.382	**1.314**	0.908	0.830	0.912	**0.818**	**0.864**	0.705	0.790	**1.428**	**0.790**
17	25	24	0.538	0.745	0.631	0.810	0.862	0.172	1.046	0.824	0.826	0.671	**0.702**	0.844	0.728	0.398	1.209	0.630
18	27	27	0.349	0.897	**0.293**	**0.822**	0.471	0.442	0.541	1.211	0.639	0.877	**0.384**	0.974	0.148	0.604	0.454	1.027
19	30	32	0.428	0.872	**0.656**	0.944	0.552	0.129	0.807	0.399	0.788	0.859	**0.810**	0.983	0.203	0.512	0.754	0.778
20	28	32	0.913	0.907	**0.671**	**0.789**	0.903	0.372	1.050	0.963	0.932	0.916	**0.799**	**0.899**	0.486	0.492	0.390	0.827
21	33	23	0.749	0.786	**0.629**	**0.488**	0.878	0.284	1.163	0.642	0.673	0.790	**0.663**	**0.592**	0.510	0.102	0.915	0.211

Supplementary table S1. Continued.

	Foretarsus1 length				Hindfemur length				Hindfemur width				Hindtibia length			
	r^2		*β*		*r^2*		*β*		*r^2*		*β*		*r^2*		*β*	
Line	♂	♀	♂	♀	♂	♀	♂	♀	♂	♀	♂	♀	♂	♀	♂	♀
1	0.838	0.855	0.984	1.181	0.966	0.672	0.970	0.850	0.745	0.485	1.146	0.911	0.975	0.608	**0.845**	0.841
2	0.754	0.682	**0.796**	0.730	0.904	0.913	**0.775**	**0.616**	0.646	0.452	1.064	1.019	0.924	0.893	**0.727**	**0.545**
3	0.829	0.696	**0.729**	1.175	0.868	0.833	0.933	**0.802**	0.651	0.083	1.189	0.207	0.857	0.795	0.908	**0.745**
4	0.491	0.090	0.630	0.420	0.848	0.480	**0.557**	**0.450**	0.387	0.032	0.823	0.315	0.850	0.544	**0.541**	**0.508**
5	0.901	0.719	**1.157**	1.032	0.811	0.295	0.920	**0.753**	0.709	0.057	**1.257**	0.373	0.758	0.233	**0.883**	0.873
6	0.681	0.819	**0.724**	1.029	0.681	0.841	0.923	**0.841**	0.315	0.264	0.640	0.759	0.657	0.868	0.839	**0.858**
7	0.595	0.778	0.972	**1.342**	0.901	0.703	0.910	0.958	0.558	0.272	1.061	**0.384**	0.913	0.709	0.924	0.916
8	0.716	0.604	**0.769**	**0.558**	0.839	0.809	0.971	0.789	0.730	0.411	1.152	0.658	0.845	0.795	0.910	0.737
9	0.746	0.852	**0.455**	0.904	0.844	0.900	1.020	0.802	0.402	0.133	1.250	-0.236	0.872	0.918	0.951	0.899
10	0.819	0.845	**0.838**	**1.504**	0.919	0.588	**0.839**	1.106	0.715	0.490	1.073	1.495	0.929	0.477	**0.842**	1.009
11	0.584	0.717	1.055	1.082	0.825	0.507	**0.794**	**0.637**	0.729	0.497	**2.006**	1.096	0.849	0.467	**0.858**	**0.773**
12	0.889	0.738	1.010	0.850	0.530	0.687	0.956	0.839	0.443	0.374	1.003	0.652	**0.482**	0.669	0.889	**0.812**
13	0.806	0.805	**0.800**	0.905	0.587	0.866	**0.801**	**0.717**	0.312	0.355	0.723	0.530	0.631	0.897	**0.762**	**0.819**
14	0.558	0.862	0.716	0.985	0.723	0.945	**0.776**	**0.868**	0.308	0.330	0.369	0.607	0.701	0.949	**0.757**	**0.811**
15	0.636	0.835	0.882	**1.211**	0.716	0.292	**0.814**	0.921	0.727	0.167	**1.305**	**1.596**	0.804	0.298	**0.840**	0.938
16	0.680	0.877	0.901	**1.219**	0.925	0.937	0.907	0.898	0.586	0.316	**1.732**	0.423	0.897	0.934	0.922	**0.829**
17	0.556	0.406	0.826	0.713	0.399	0.276	0.956	0.716	0.401	0.064	2.051	0.005	0.367	0.285	0.893	0.814
18	0.249	0.826	0.420	1.156	0.681	0.919	**0.443**	0.998	0.569	0.507	1.423	1.348	0.729	0.943	**0.590**	1.033
19	0.691	0.701	1.033	1.098	0.862	0.852	0.885	0.893	0.317	0.290	0.891	1.114	0.855	0.883	0.871	**0.862**
20	0.603	0.827	0.787	**1.223**	0.951	0.887	0.992	0.992	0.381	0.409	0.451	**0.639**	0.942	0.931	0.874	0.971
21	0.648	0.650	**0.768**	0.775	0.397	0.780	**0.877**	**0.521**	0.520	0.369	**1.441**	1.145	0.396	0.855	**0.877**	**0.732**

Supplementary table S1. Continued.

	Hindtibia width				Hindtarsus1 length				Wing length				Wing width			
	r^2		β		r^2		β		r^2		β		r^2		β	
Line	♂	♀	♂	♀	♂	♀	♂	♀	♂	♀	♂	♀	♂	♀	♂	♀
1	0.765	0.489	0.692	**0.613**	0.942	0.660	1.068	1.042	0.933	0.735	**0.617**	**0.617**	0.859	0.788	**0.562**	**0.552**
2	0.398	0.398	**0.449**	**0.417**	0.907	0.645	0.911	**0.676**	0.678	0.803	**0.577**	**0.456**	0.447	0.724	**0.519**	**0.472**
3	0.668	0.340	0.780	**0.462**	0.829	0.798	1.129	1.142	0.948	0.810	**0.633**	**0.588**	0.938	0.721	**0.706**	**0.591**
4	0.499	0.000	0.382	**-0.019**	0.827	0.274	0.729	0.664	0.826	0.147	**0.363**	0.206	0.694	0.181	**0.613**	0.219
5	0.583	0.008	0.951	0.426	0.760	0.273	**1.208**	1.073	0.914	0.837	**0.624**	**0.666**	0.813	0.715	**0.467**	**0.488**
6	0.403	0.371	**0.479**	**0.584**	0.686	0.843	1.044	1.028	0.832	0.844	**0.520**	**0.590**	0.844	0.791	**0.558**	**0.564**
7	0.485	0.276	0.826	**0.427**	0.850	0.711	1.133	**1.271**	0.755	0.379	**0.488**	**0.661**	0.764	0.500	**0.445**	**0.533**
8	0.693	0.456	0.715	0.632	0.820	0.820	1.102	0.990	0.844	0.757	**0.590**	**0.507**	0.838	0.597	**0.614**	**0.435**
9	0.335	0.457	0.575	0.927	0.783	0.709	1.171	0.610	0.685	0.858	**0.596**	0.748	0.554	0.587	0.697	0.674
10	0.671	0.568	**0.790**	0.990	0.922	0.305	1.015	1.058	0.964	0.969	**0.707**	**0.809**	0.927	0.947	**0.755**	**0.787**
11	0.706	0.335	1.173	0.708	0.769	0.344	1.192	0.902	0.745	0.702	**0.520**	**0.515**	0.783	0.666	**0.626**	**0.528**
12	0.431	0.282	0.810	**0.479**	0.420	0.402	1.124	**0.729**	0.966	0.887	**0.691**	**0.656**	0.896	0.719	**0.600**	**0.641**
13	0.547	0.470	**0.625**	**0.637**	0.615	0.861	1.177	0.984	0.917	0.843	**0.555**	**0.574**	0.503	0.636	**0.557**	**0.445**
14	0.265	0.352	0.330	0.498	0.699	0.894	0.849	0.861	0.868	0.837	**0.437**	**0.538**	0.846	0.757	**0.573**	**0.489**
15	0.602	0.289	0.804	0.966	0.750	0.296	1.081	1.172	0.831	0.829	**0.599**	**0.579**	0.696	0.765	**0.534**	**0.685**
16	0.705	0.257	1.059	**0.448**	0.807	0.866	1.124	1.058	0.758	0.903	**0.617**	**0.592**	0.723	0.839	**0.535**	**0.587**
17	0.443	0.106	1.227	0.304	0.303	0.344	0.940	1.050	0.834	0.717	0.776	**0.614**	0.840	0.473	0.883	**0.595**
18	0.483	0.748	0.679	1.020	0.548	0.865	**0.585**	**1.253**	0.573	0.916	**0.354**	**0.693**	0.615	0.834	**0.402**	**0.613**
19	0.269	0.233	**0.468**	**0.438**	0.630	0.683	0.843	0.973	0.515	0.832	**0.605**	**0.658**	0.402	0.583	**0.578**	**0.572**
20	0.641	0.574	0.736	0.849	0.921	0.903	1.159	**1.126**	0.961	0.808	**0.694**	**0.745**	0.932	0.722	**0.665**	**0.701**
21	0.493	0.472	0.934	0.844	0.407	0.743	1.050	0.609	0.671	0.667	**0.521**	**0.441**	0.618	0.510	**0.601**	**0.540**

Supplementary figure S1. Sex-specific static allometries for the 21 genotypes and sexes. Horizontal dashed lines indicate isometry. The vertical error bars represent 95% confidence intervals. That there was more variation in slopes for leg width compared to length dimensions was likely the result of greater measurement error due to less well-defined landmarks for the much shorter distances to measure.

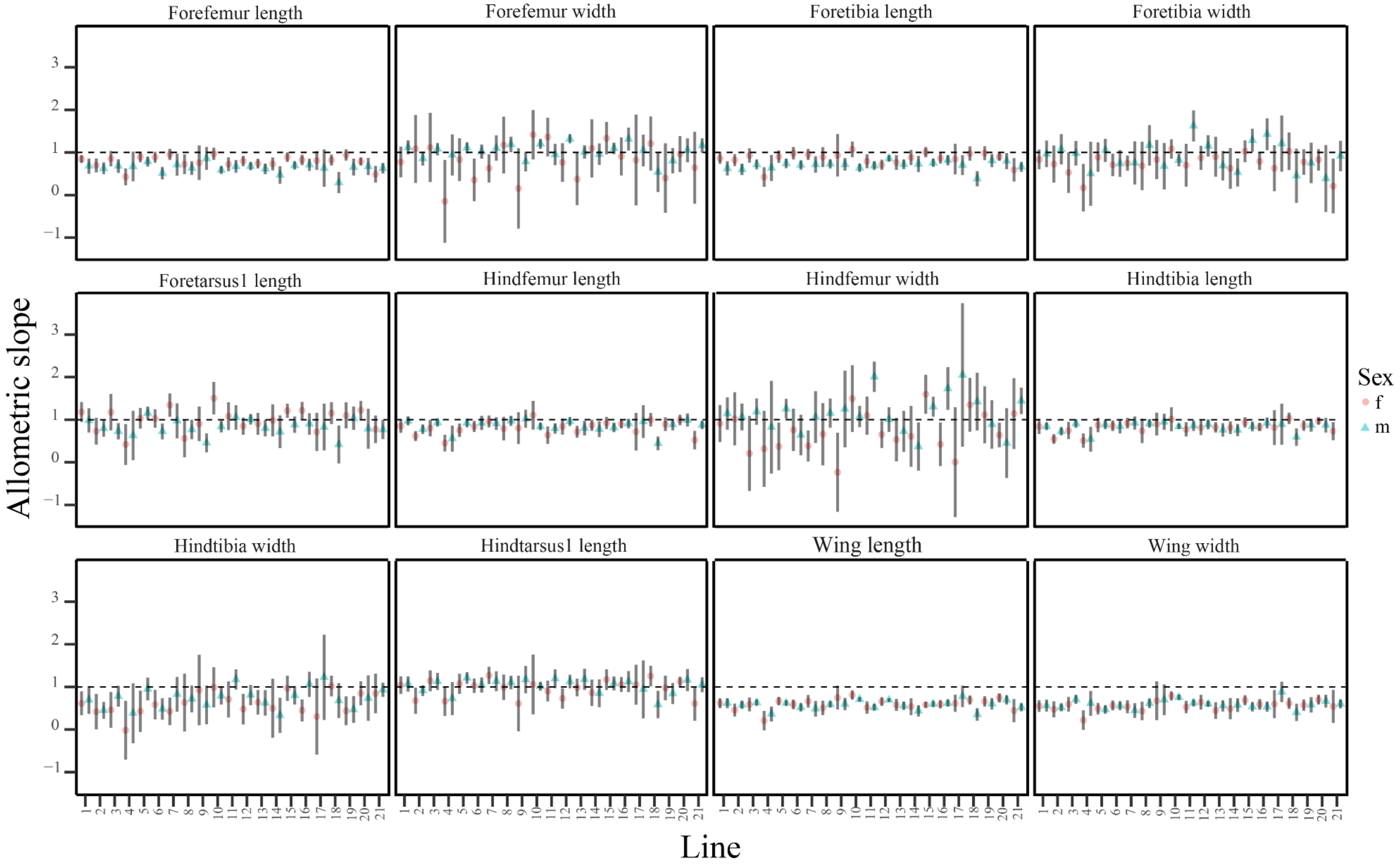

Supplement: Supplementary file 1 — Supplementary Material 1 [file 10682_2022_10226_MOESM1_ESM.pdf]
